# Hypoxia promotes liver-stage malaria infection in primary human hepatocytes *in vitro*

**DOI:** 10.1242/dmm.013490

**Published:** 2013-11-28

**Authors:** Shengyong Ng, Sandra March, Ani Galstian, Kirsten Hanson, Tânia Carvalho, Maria M. Mota, Sangeeta N. Bhatia

**Affiliations:** 1 Department of Biological Engineering, Massachusetts Institute of Technology, Cambridge, MA 02139, USA.; 2 Health Sciences and Technology, Institute for Medical Engineering and Science, Massachusetts Institute of Technology, Cambridge, MA 02139, USA.; 3 Broad Institute, Cambridge, MA 02142, USA.; 4 Unidade de Malária, Instituto de Medicina Molecular, Universidade de Lisboa, 1649-028 Lisboa, Portugal.; 5 Howard Hughes Medical Institute, Koch Institute, and Electrical Engineering and Computer Science, Massachusetts Institute of Technology, Cambridge, MA 02139, USA.; 6 Department of Medicine, Brigham and Women’s Hospital, Boston, MA 02115, USA.

**Keywords:** Hypoxia, Primary hepatocytes, Liver-stage malaria

## Abstract

Homeostasis of mammalian cell function strictly depends on balancing oxygen exposure to maintain energy metabolism without producing excessive reactive oxygen species. *In vivo*, cells in different tissues are exposed to a wide range of oxygen concentrations, and yet *in vitro* models almost exclusively expose cultured cells to higher, atmospheric oxygen levels. Existing models of liver-stage malaria that utilize primary human hepatocytes typically exhibit low *in vitro* infection efficiencies, possibly due to missing microenvironmental support signals. One cue that could influence the infection capacity of cultured human hepatocytes is the dissolved oxygen concentration. We developed a microscale human liver platform comprised of precisely patterned primary human hepatocytes and nonparenchymal cells to model liver-stage malaria, but the oxygen concentrations are typically higher in the *in vitro* liver platform than anywhere along the hepatic sinusoid. Indeed, we observed that liver-stage *Plasmodium* parasite development *in vivo* correlates with hepatic sinusoidal oxygen gradients. Therefore, we hypothesized that *in vitro* liver-stage malaria infection efficiencies might improve under hypoxia. Using the infection of micropatterned co-cultures with *Plasmodium berghei*, *Plasmodium yoelii* or *Plasmodium falciparum* as a model, we observed that ambient hypoxia resulted in increased survival of exo-erythrocytic forms (EEFs) in hepatocytes and improved parasite development in a subset of surviving EEFs, based on EEF size. Further, the effective cell surface oxygen tensions (pO_2_) experienced by the hepatocytes, as predicted by a mathematical model, were systematically perturbed by varying culture parameters such as hepatocyte density and height of the medium, uncovering an optimal cell surface pO_2_ to maximize the number of mature EEFs. Initial mechanistic experiments revealed that treatment of primary human hepatocytes with the hypoxia mimetic, cobalt(II) chloride, as well as a HIF-1α activator, dimethyloxalylglycine, also enhance *P. berghei* infection, suggesting that the effect of hypoxia on infection is mediated in part by host-dependent HIF-1α mechanisms.

## INTRODUCTION

Malaria affects 250 million people and causes approximately a million deaths each year (World Health Organization, 2011). The liver stage of malaria is an attractive target for the development of antimalarial drugs and vaccines (Prudêncio et al., 2006; Mazier et al., 2009), especially with the goal of malaria eradication, but is relatively poorly understood. *In vitro* models that recapitulate the liver stages of human malaria are needed to identify compounds that have potential antimalarial activity, but most of these models are dependent on cell lines (Gego et al., 2006; Meister et al., 2011) due to limitations in *in vitro* culture of primary adult hepatocytes. There is evidence that mimicking the *in vivo* hepatic microenvironment, such as by adding cell-cell interactions, cell-matrix interactions and controlling tissue microarchitecture, can improve *in vitro* models of the liver (Dunn et al., 1989; Sivaraman et al., 2005; Khetani and Bhatia, 2008; Kidambi et al., 2009). For example, micropatterned co-cultures (MPCCs) of primary human hepatocytes (PHHs) and supporting stromal fibroblasts result in stable hepatocyte function, including albumin secretion, urea production and cytochrome P450 levels, for several weeks compared with hepatocytes alone (Khetani and Bhatia, 2008). Another feature of the *in vivo* hepatic microenvironment is the presence of a range of oxygen tensions (Wölfle et al., 1983), which is thought to be a factor that contributes to hepatic zonation, a compartmentalization of functions along the axis of perfusion (Jungermann and Kietzmann, 1996; Jungermann and Kietzmann, 2000). Previous studies have shown that exposing mixed populations of primary rat hepatocytes to physiological gradients of oxygen tension can induce compartmentalization *in vitro*, render the cells selectively susceptible to zonal hepatotoxins (Allen and Bhatia, 2003; Allen et al., 2005) and recapitulate the zonated patterns of carbohydrate-metabolizing enzyme gene expression *in vitro* (Wölfle et al., 1983; Jungermann and Kietzmann, 1996; Kietzmann and Jungermann, 1997). Thus, *in vitro* liver-stage malaria culture platforms might be improved by altering microenvironmental oxygen concentrations.

Ambient oxygen concentrations have a broad spectrum of biological impact, influencing diverse pathways from homeostasis to development (Semenza, 2011). The role of oxygen has been explored in a range of infectious diseases. For instance, hyperoxia reduces certain bacterial and *Apicomplexan* infections *in vitro* or *in vivo* (Park et al., 1992; Tsuneyoshi et al., 2001; Arrais-Silva et al., 2006), whereas hypoxia promotes hepatitis C virus infection *in vitro* (Vassilaki et al., 2013) and *Trypanosoma lewisi* infections *in vivo* (Hughes and Tatum, 1956b). In the malaria field, previous studies have probed the effect of atmospheric oxygen on parasitemia in rodent and avian disease models. In particular, *Plasmodium berghei*-infected rats or *Plasmodium cathemerium*-infected canaries subjected to hypoxia exhibited increased levels of parasitemia (Hughes and Tatum, 1955; Hughes and Tatum, 1956a), whereas hyperoxia decreased *P. berghei* parasitemia (Rencricca et al., 1981; Blanco et al., 2008) and prevented early death caused by experimental cerebral malaria in the *P. berghei*-ANKA mouse model (Blanco et al., 2008). Furthermore, continuous *in vitro* culture of the blood stages of *Plasmodium falciparum* was first achieved by reducing atmospheric oxygen levels (Trager and Jensen, 1976), and subsequent studies have characterized this microaerophilic nature of blood stage *P. falciparum* (Torrentino-Madamet et al., 2011).

**TRANSLATIONAL IMPACT**

**Clinical issue**

Malaria is a mosquito-borne parasitic disease that impacts millions of people worldwide and that causes about a million deaths a year. Several different *Plasmodium* parasites cause malaria but all have a complex life cycle. *Plasmodium* parasites enter the human body as sporozoites, which travel to the liver where they develop and grow without causing any symptoms. After a few days, merozoites are released from the liver cells and invade red blood cells. Here they replicate rapidly, before bursting out and invading more red blood cells. The recurring flu-like symptoms and more serious complications of malaria such as anemia and organ damage are caused by this cyclical increase in the parasitic burden.

**Results**

The liver stage of the *Plasmodium* life-cycle is an attractive target for drug treatment. However, to develop drugs that attack this stage, model systems that mimic normal human liver responses to parasitic infection are needed. Existing models are generally hard to infect, in part because cells that are grown in tissue culture are generally exposed to higher levels of oxygen than they would experience inside the body. In this study, the authors first show that the growth of malaria parasites in the livers of infected mice correlates with the natural variation in oxygen levels within the liver. They then show that the exposure of micropatterned co-cultures of primary human hepatocytes and supporting stromal cells to different levels of oxygen leads to profound changes in malaria infection efficiency and parasite development. Finally, they report that the effect of different oxygen levels on the infection of liver cells by malaria parasites is partly due to the activation of the HIF-1α intracellular oxygen sensing signaling pathway.

**Implications and future directions**

These findings, in combination with existing literature on the impact of oxygen levels on the maintenance of *in vivo*-like hepatocyte functions *in vitro*, demonstrate that optimization of the oxygen levels experienced by human liver cells grown in tissue culture is needed to maximize malaria infection rates. This new information can now be used to develop improved models of liver-stage malaria for antimalarial drug development. The study also identifies one oxygen-dependent host mechanism that influences liver-stage malaria parasite development. Future studies on this mechanism and on other oxygen-dependent host or parasite mechanisms that might potentially affect the liver stage of *Plasmodium* development should further facilitate antimalarial drug development.

In this study, we explored the influence of cell surface oxygen on liver-stage malaria infection of PHHs. We used an *in vitro* model of hepatocyte culture that is phenotypically stable, responsive to ambient oxygen and supports the liver stage of malaria (March et al., 2013). Using this model system and a mathematical framework to estimate the cell surface oxygen partial pressures (pO_2_) under a variety of experimental manipulations, we show that oxygen has a profound impact on the *Plasmodium* liver stage. In particular, both infection efficiency and development of exo-erythrocytic forms (EEFs) can be perturbed by altering cell surface oxygen concentrations. We identified an optimal cell surface oxygen level for maximizing infection and demonstrate that host HIF-1α is at least partially responsible for this response.

## RESULTS

### *In vivo* EEF development correlates with hepatic oxygen gradients

**Fig. 1.**
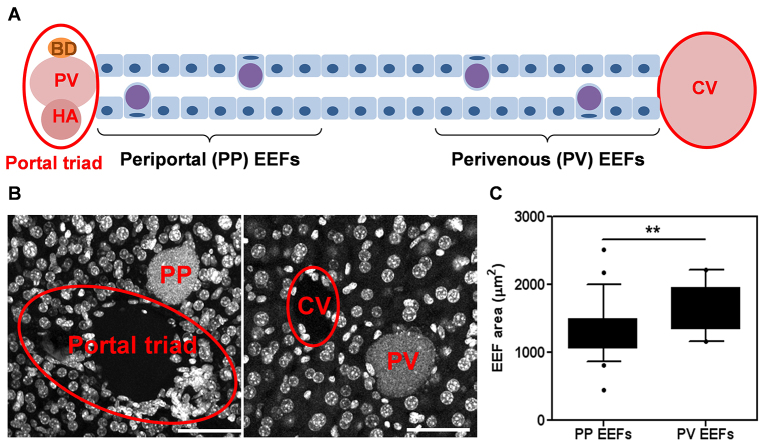
***Plasmodium* EEF development correlates with hepatic oxygen gradients *in vivo***. (A) Schematic of liver sinusoid denoting the definition of periportal (PP) EEFs and perivenous (PV) EEFs used for EEF size quantification. (B) 50-μm liver slices were stained with DAPI, and confocal *z*-stacks were made of GFP-expressing *P. yoelii* EEFs within 8 hepatocyte lengths of either the portal triad (periportal) or the central vein (perivenous) for which the maximal *XY* area could be determined within the slice. (C) Maximal *XY* areas of *P. yoelii* perivenous or periportal EEFs (as defined for A) at 46 hours post-infection in murine liver; **P<0.01, two-tailed *t*-test. Scale bar: 50 μm. PV, portal vein; BD, bile duct; HA, hepatic artery; CV, central vein.

Oxygen tensions in the hepatic sinusoids vary from 30–75 mmHg between the perivenous and periportal regions, respectively (Wölfle et al., 1983). To investigate whether this variation in oxygen concentration exerts an influence on liver-stage *Plasmodium* infection *in vivo*, C57BL/6 mice were infected with GFP-expressing *Plasmodium yoelii* sporozoites, a host-parasite combination that supports robust liver-stage infection (Douradinha et al., 2007), and their livers collected 46 hours post-infection. Two populations of *P. yoelii* EEFs were defined to test the hypothesis that the hepatic sinusoidal variation of oxygen concentration correlates with EEF growth. EEFs were defined as periportal EEFs if they were found within eight cell-lengths of the hepatic portal triad, and perivenous EEFs if they were found within eight cell-lengths of the hepatic central vein (Fig. 1A). This definition minimizes the likelihood of an EEF being simultaneously defined as periportal and perivenous, taking into consideration that the number of hepatocytes between the portal triad and the central vein of a mouse liver is ~20. Immunohistochemical analysis of infected liver sections (Fig. 1B) revealed that the maximal size of perivenous *P. yoelii* EEFs were significantly larger than periportal *P. yoelii* EEFs (Fig. 1C), suggesting that oxygen concentrations could be a parameter that influences liver stage *Plasmodium* infection of primary hepatocytes *in vitro*.

### Ambient hypoxia increases survival and growth of liver-stage malaria parasites in PHH MPCCs

**Fig. 2.**
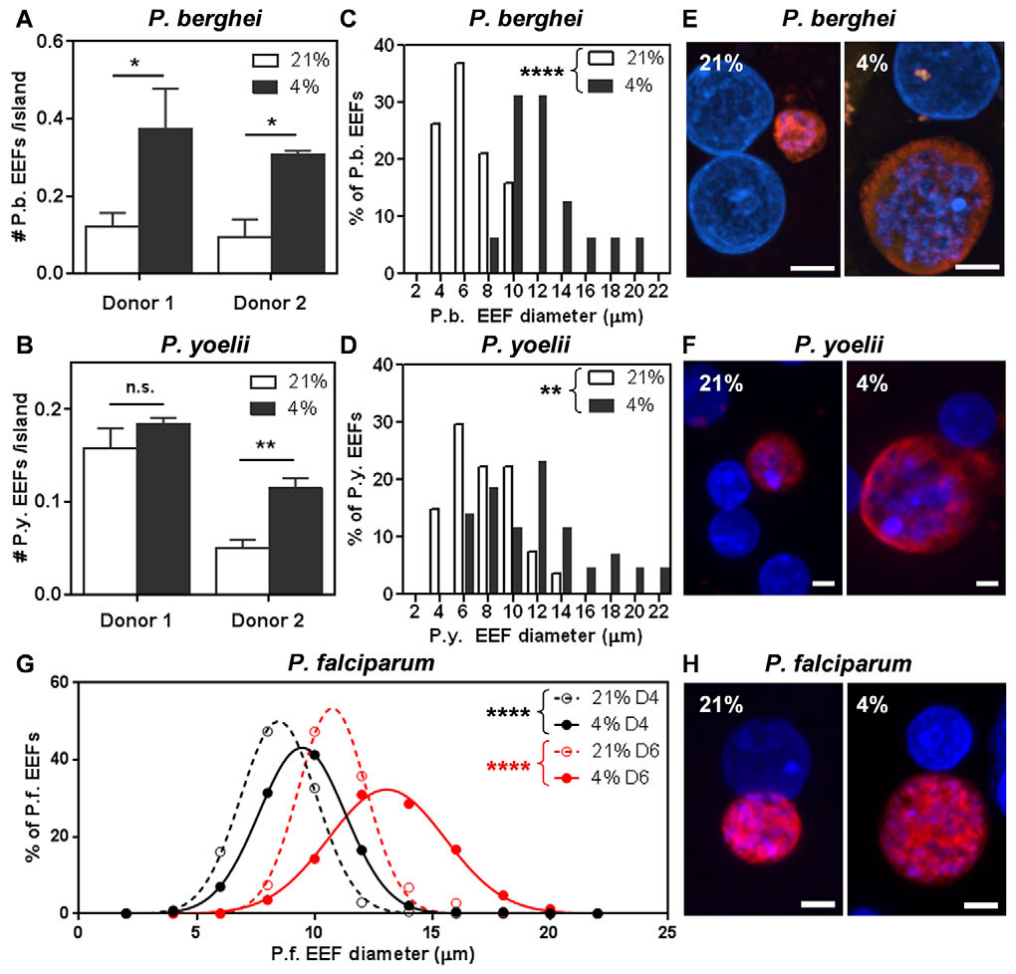
**Ambient hypoxia increases liver-stage malaria infection *in vitro***. (A,B) Ambient hypoxia (4% O_2_) increases the number of *P. berghei* and *P. yoelii* EEFs in PHH MPCCs at 48 hours post-infection. (C,D,G) Ambient hypoxia (black symbols or bars, 4% O_2_) increases the EEF size distribution of *P. berghei* and *P. yoelii* at 48 hours post-infection and *P. falciparum* at 4 and 6 days post-infection in PHH MPCCs compared with normoxia (white symbols or bars, 21% O_2_). (E,F,H) Representative immunofluorescence images of *P. berghei*, *P. yoelii* EEFs at 48 hours post-infection, and *P. falciparum* EEFs at 6 days post-infection at either ambient 21% or 4% O_2_. EEFs were stained for *Plasmodium* HSP70 (clone 2E6 for *P. berghei* and *P. yoelii*, clone 4C9 for *P. falciparum*). Scale bars: 5 μm. *P<0.05, **P<0.01, ****P<0.0001; two-tailed *t*-test.

To investigate whether hypoxia influences *P. berghei* infection of human liver cells *in vitro*, micropatterned co-cultures of primary human hepatocytes and supporting stromal fibroblasts were maintained at 4% O_2_ for 24 hours before infection. A 3-hour exposure to *P. berghei* sporozoites was followed by an additional 48 hours of hypoxic culture, at which point infection efficiency was determined based on *Plasmodium* HSP70 immunofluorescence. The number of *P. berghei* EEFs per hepatocyte island was elevated in response to hypoxic incubation of PHHs before, during and after infection (Fig. 2A). A significant upward shift in the size distribution of *P. berghei* EEFs in hypoxic cultures compared to normoxic cultures was also observed (Fig. 2C,E). This pattern of improved infectivity was observed in more than one lot of cryopreserved PHHs (Fig. 2A; supplementary material Fig. S1A) and also in HepG2 cells (supplementary material Fig. S1C). Hypoxia-treated hepatocytes exhibited a similar increase in susceptibility to *P. yoelii* infection (Fig. 2B,D,F; supplementary material Fig. S1B), suggesting that the observed effect of hypoxia is not restricted to a particular *Plasmodium* spp.

Because *P. berghei* liver-stage infections mature at 55–65 hours post-infection *in vitro* (Graewe et al., 2011), *P. berghei* EEF sizes were quantified at 56 hours and 65 hours post-infection to address the possibility that hypoxia could be speeding up parasite development instead of increasing the potential for parasite growth. *P. berghei* EEFs were larger in hypoxic cultures at 48, 56 and 65 hours post-infection (supplementary material Fig. S1F). Furthermore, the number of *P. berghei* EEFs per hepatocyte island was consistently higher in hypoxic cultures at 48, 56 and 65 hours post-infection (supplementary material Fig. S1E). Given that both EEF numbers and sizes are larger in hypoxic cultures throughout the late liver stages of *P. berghei* infection, this suggests that the total number of potential merozoites is larger under hypoxia than under normoxia. Consistent with this prediction, the number of nuclei in *P. berghei* EEFs at 65 hours post-infection was significantly higher in hypoxic cultures compared with the normoxic control (supplementary material Fig. S1H). *P. berghei* EEFs were also able to develop normally under hypoxia, as shown by the expression of the mid-liver-stage marker, PbMSP-1, at 65 hours post-infection and the appearance of various EEF morphologies characteristic of late liver-stage EEFs (supplementary material Fig. S2). Moreover, the percentage of MSP1-positive *P. berghei* EEFs was significantly higher in hypoxic cultures at 56 and 65 hours post-infection (supplementary material Fig. S1G), suggesting that the EEFs progress into the later phases of the liver stage more successfully under hypoxia.

Importantly, the effect of hypoxia on EEF size translated to the human *Plasmodium* species *P. falciparum*, as shown by the finding that ambient hypoxia increased the size of *P. falciparum* EEFs in hepatocytes at both 4 and 6 days post-infection (Fig. 2G,H). However, the number of *P. falciparum* EEFs did not increase in hypoxic cultures maintained at 4% O_2_ (supplementary material Fig. S1D).

### Optimization of cell surface oxygen tension for *in vitro* liver-stage malaria infection

**Fig. 3.**
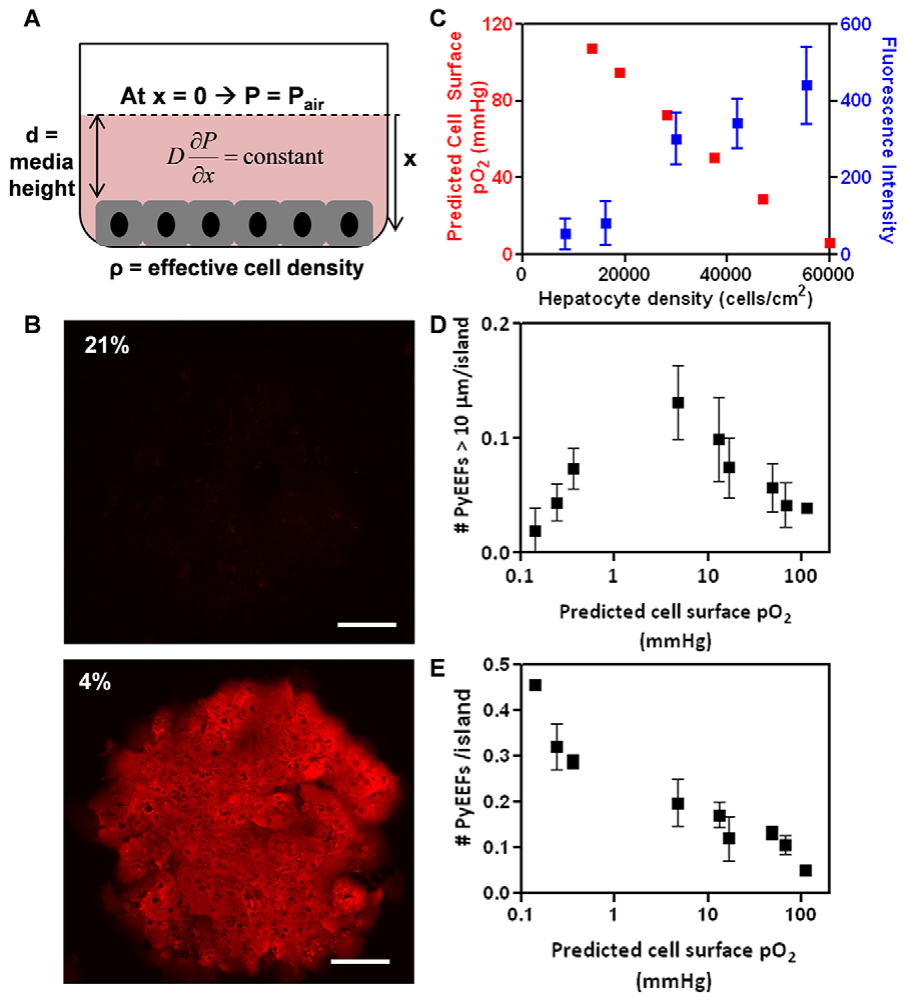
**Optimal pO_2_ exists for development of mature *Plasmodium* EEFs.** (A) Schematic of steady-state diffusion-reaction model with three parameters that determine cell surface oxygen concentration: atmospheric pO_2_ (*P*_air_), height of medium and cell density. (B) Validation of effect of atmospheric pO_2_ on cell surface pO_2_ by Hypoxyprobe™ staining. Hypoxyprobe™ forms covalent adducts with thiol groups at pO_2_<10 mmHg. (C) Modulation of cell surface pO_2_ by varying effective cell density as predicted by the model (red), and Hypoxyprobe™ fluorescence intensity (blue). (D,E) Modulation of cell surface pO_2_ by simultaneously varying both atmospheric pO_2_ and effective cell density results (D) in a biphasic relationship between the number of well-developed *P. yoelii* EEFs and the predicted cell surface pO_2_ and (E) in a monotonic relationship between the total number of *P. yoelii* EEFs versus predicted cell surface pO_2_ in PHH MPCCs at 48 hours post-infection. Scale bars: 100 μm.

Given the observed impact of prolonged exposure to a reduced oxygen concentration, we sought an optimal set of conditions that might maximize the elevated infection of PHHs. By applying a mathematical model of diffusion and reaction solved at steady-state conditions (Yarmush et al., 1992) to PHH MPCCs (Fig. 3A; supplementary material Fig. S3), it was estimated that the typical cell surface pO_2_ when cultures are incubated at normoxia ranges from 110 to 130 mmHg (Table 1). In contrast, *in vivo* blood pO_2_ (not at the cell surface) ranges from 30 to 75 mmHg in the hepatic sinusoid (Wölfle et al., 1983). Therefore, culture at ambient hypoxia could improve liver-stage malaria infection by reducing cell surface pO_2_ to a more physiologically relevant level. To test this hypothesis, a Hypoxyprobe™ assay that incorporates a hypoxic marker, pimonidazole hydrochloride (Varghese et al., 1976), was conducted to compare the cell surface pO_2_ in PHHs incubated at either normoxia or ambient hypoxia. Consistent with our hypothesis, incubation of PHHs at ambient hypoxia results in an increase in Hypoxyprobe™ staining relative to normoxia-cultured MPCCs (Fig. 3B), confirming that ambient hypoxia indeed results in a decrease in cell surface pO_2_ experienced by the hepatocytes.

**Table 1.**
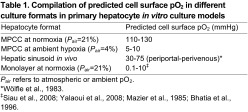
Compilation of predicted cell surface pO_2_ in different culture formats in primary hepatocyte *in vitro* culture models

*P*_air_ refers to atmospheric or ambient pO_2_.

* Wölfle et al., 1983.

‡ Siau et al., 2008; Yalaoui et al., 2008; Mazier et al., 1985; Bhatia et al., 1996.

Cell surface pO_2_ of MPCCs can also be altered by modifying parameters such as media height and hepatocyte density (Fig. 3A). The model predicts that cell surface pO_2_ decreases as media height increases (supplementary material Fig. S3B). Indeed, elevating the media height in wells of normoxic cultures resulted in an increase in Hypoxyprobe™ staining at the cell surface (supplementary material Fig. S4A,B). The greater media height also led to increased numbers of *P. berghei* EEFs at 48 hours post-infection (supplementary material Fig. S4C), collectively supporting the hypothesis that the effects of ambient hypoxia on *in vitro* liver-stage malaria infection efficiencies are mediated by a decrease in the effective cell surface pO_2_ experienced by the hepatocytes.

Modeling also predicts that cell surface pO_2_ will decrease as cell density increases (Fig. 3C; supplementary material Fig. S2A). However, modifications to hepatocyte density in a conventional monolayer culture might also influence infection efficiency due to the resulting changes in hepatocyte survival, polarization and morphology, rather than in response to changes in cell surface pO_2_. To vary hepatocyte density while preserving the homotypic interactions necessary for hepatocyte survival and functional maintenance, the density of the hepatocyte island patterning was varied in MPCCs. These modifications led to perturbations of the cell surface pO_2_ as predicted by the model, based on Hypoxyprobe™ staining results (Fig. 3C). The simultaneous variation of both hepatocyte island density and atmospheric oxygen level permits fine-tuning of cell surface oxygen levels that span four orders of magnitude (supplementary material Table S1). Infections with *P. yoelii* across this range of conditions yield a monotonic increase in total EEFs as cell surface pO_2_ decreases (Fig. 3E). However, a threshold cell surface pO_2_ is observed at 5–10 mmHg, below which the number of mature EEFs (diameter >10 μm) decreases as cell surface pO_2_ declines (Fig. 3D). This biphasic relationship between the number of mature EEFs and cell surface pO_2_ suggests that there is an optimal cell surface pO_2_ for maximizing the number of mature EEFs in infected MPCCs. The combination of the optimal hepatocyte island density under ambient hypoxia (4% O_2_), which gives rise to the optimal cell surface pO_2_ of 5–10 mmHg was hence used for subsequent experiments.

### Kinetics of hypoxic treatment alters liver-stage malaria infection *in vitro*

**Fig. 4.**
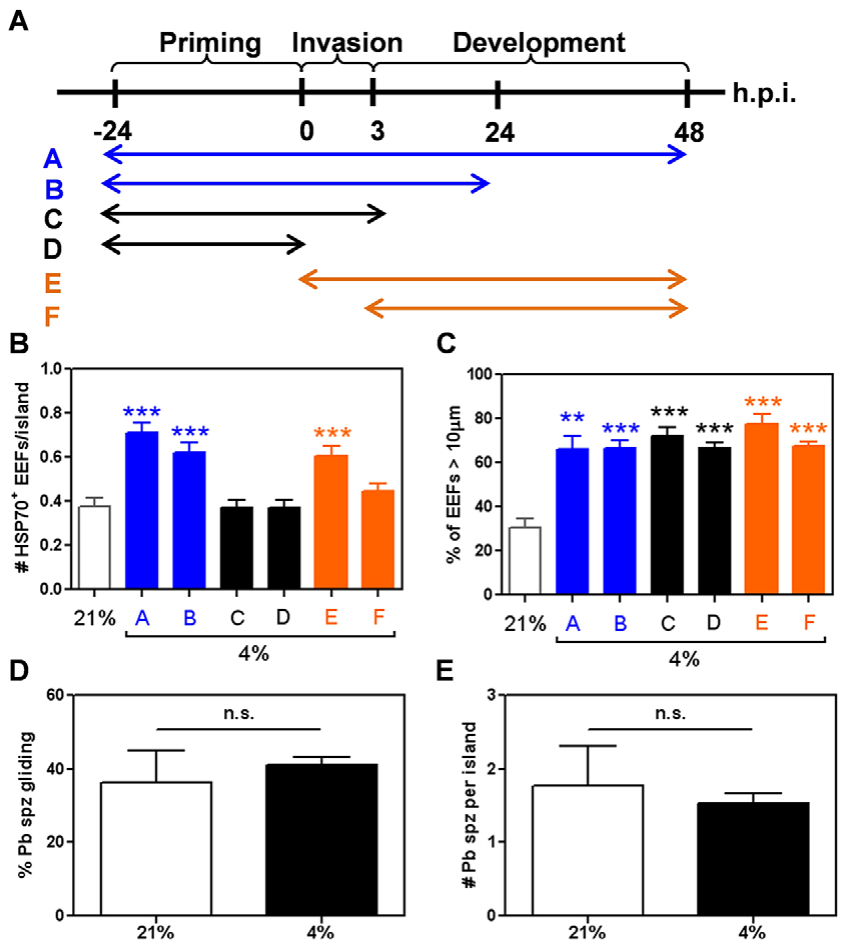
**Kinetics of hypoxic treatment alters liver-stage malaria infection *in vitro***. (A) Schematic of differential hypoxia treatment regimes. (B) Effect of differential hypoxia kinetic regimes on the number of *P. berghei* EEFs at 48 hours post-infection. (C) Effect of differential hypoxia kinetic regimes on *P. berghei* EEF sizes at 48 hours post-infection. (D) Effect of ambient hypoxia on *P. berghei* sporozoite gliding. (E) Effect of ambient hypoxia on *P. berghei* sporozoite entry into hepatocytes at 3 hours post-infection. **P<0.01, ***P<0.001; one way ANOVA with Tukey’s multiple comparison test.

The hypoxia experiments performed thus far had exposed the PHH MPCCs to hypoxia throughout the 24 hours before infection, during infection (0–3 hours) and after infection (3–48 hours), termed the priming, invasion and development phases, respectively. To assay whether improved infectivity requires each of these three phases of hypoxic treatment, MPCCs were incubated at ambient hypoxia over varying portions of the assay (Fig. 4A). Increased numbers of EEFs at 48 hours post-infection were only observed when the infected MPCCs were cultured under hypoxia during the invasion and development phases (Fig. 4B, conditions A, B, E). In contrast, MPCCs pre-treated with hypoxia before infection and subsequently returned to normoxia (Fig. 4B, conditions C, D) did not exhibit an increase in EEF number. These findings suggest that hypoxia treatment improves late-stage infection rates by reducing the attrition rate of EEFs rather than promoting the initial susceptibility of the host hepatocytes to sporozoite invasion. However, hypoxia over varying portions of the assay did not change the proportion of large EEFs 48 hours post-infection (Fig. 4C).

### Hypoxia does not increase sporozoite-dependent or host-dependent invasion

To examine whether the hypoxia-mediated change in hepatocyte infectivity stems from an impact on sporozoite function, sporozoite gliding motility and sporozoite entry into hepatocytes were assayed. Ambient hypoxia did not result in a significant difference in the gliding motility of *P. berghei* sporozoites (Fig. 4D), and hypoxic treatment of hepatocytes did not change the number of the sporozoites that successfully entered hepatocytes (Fig. 4E), suggesting that hypoxia does not improve late-stage infection efficiencies via sporozoite or host-mediated increases in the initial invasion rate, but rather by affecting the ability of the host cell to support EEF survival and growth.

### Host HIF-1α induction promotes EEF survival in infected hepatocytes

**Fig. 5.**
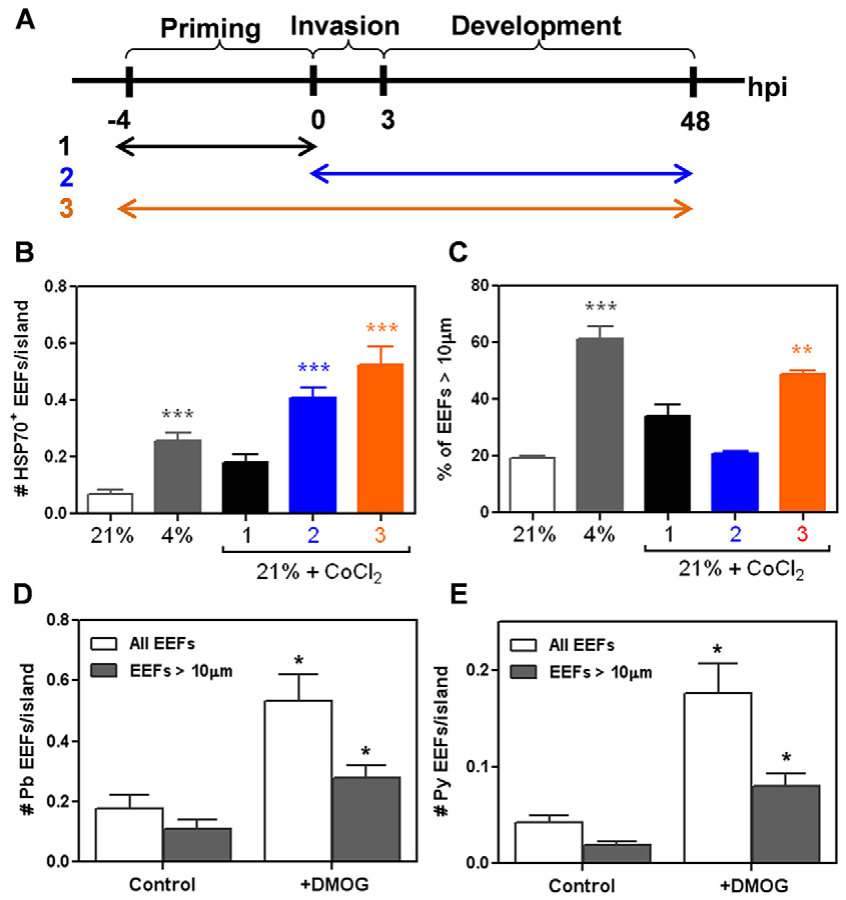
**Host HIF-1α induction increases EEF numbers in infected hepatocytes.** (A) Schematic of cobalt(II) chloride treatment of PHH MPCCs during infection with *P. berghei.* (B,C) Effect of cobalt(II) treatment of PHH MPCCs at 21% O_2_ on (B) the number of *P. berghei* EEFs at 48 hours post-infection and (C) on the percentage of *P. berghei* EEFs of >10 μm at 48 hours post-infection; **P<0.01, ***P<0.001, one way ANOVA with Tukey’s multiple comparison test. (D,E) Effect of DMOG treatment of PHH MPCCs at 21% O_2_ on (D) the numbers of *P. berghei* EEFs and (E) the number of *P. yoelii* EEFs at 48 hours post-infection; *P<0.05, two-tailed *t*-test.

The hypoxic response of mammalian cells is largely mediated by the hypoxia-inducible factor-1 (HIF-1) pathway (Semenza, 2012). Consistent with the reported literature, gene set enrichment analysis (GSEA) of PHH MPCCs incubated at ambient hypoxia revealed a marked enrichment for the expression of genes that are transcriptionally regulated by HIF-1α relative to normoxic MPCCs (supplementary material Fig. S6A). Cobalt(II) chloride is a hypoxia mimetic that has been reported to induce the intracellular stabilization of HIF-1α and lead to the transcriptional activation of downstream hypoxia-responsive genes (Jaakkola et al., 2001). To determine whether ambient hypoxia promotes liver-stage malaria infection in PHH MPCCs via host HIF-1α induction, pharmacologic activation of HIF-1α in PHH MPCCs by cobalt(II) chloride was performed at normoxia in three different combinations of the priming, invasion and development phases (Fig. 5A). Cobalt(II) treatment of PHH MPCCs at normoxia in any of the three combinations tested resulted in an increased number of *P. berghei* EEFs at 48 hours post-infection, with the greatest effect observed if cobalt(II) was present throughout all three phases of priming, invasion and development (Fig. 5B). Of note, although ambient hypoxia (4% O_2_) consistently led to the emergence of a subset of larger EEFs relative to normoxic controls, cobalt(II) treatment did not fully replicate this outcome (Fig. 5C; supplementary material Fig. S5A).

Under normoxia, HIF-1α is constitutively marked for proteasomal degradation by prolyl hydroxylase (PHD). Inhibition of PHD by a small molecule, dimethyloxalylglycine (DMOG), results in HIF-1α stabilization and the associated downstream host hypoxic responses (Jaakkola et al., 2001). GSEA of hypoxic MPCCs also shows a marked enrichment for the expression of a set of genes that are upregulated under DMOG treatment (supplementary material Fig. S6B) (Elvidge et al., 2006). Consistent with the effect of cobalt(II) treatment on *P. berghei* infection at normoxia, PHH MPCCs that were treated with DMOG at normoxia demonstrate increased numbers of *P. berghei* and *P. yoelii* EEFs at 48 hours post-infection (Fig. 5D,E), with the number of *P. berghei* EEFs increasing in a dose-dependent fashion with DMOG concentration (supplementary material Fig. S5B). However, DMOG treatment did not lead to the emergence of a subset of larger EEFs compared to the untreated control, in contrast to ambient hypoxia (supplementary material Fig. S5C). Further increases in DMOG concentration inhibited EEF development (supplementary material Fig. S5C), which is reminiscent of the effect of extremely low levels of pO_2_ on the number of well-developed EEFs (Fig. 3D). Together, these data suggest that intermediate levels of HIF-1α activation in the host hepatocyte support EEF survival but not EEF growth, and that higher levels of HIF-1α might inhibit EEF growth and mediate the biphasic effect of pO_2_ on EEF size observed in earlier experiments.

## DISCUSSION

Using an *in vitro* model of primary hepatocyte culture that stabilizes PHH function, is oxygen-responsive, and infectible with liver-stage malaria, we applied a mathematical framework to estimate cell surface oxygen tensions under a variety of experimental manipulations. We have shown that the cell surface oxygen concentration experienced by primary adult human hepatocytes *in vitro* influences their ability to support a productive liver-stage malaria infection by *P. berghei*, *P. yoelii* and *P. falciparum*. Moreover, we identified an optimal cell surface oxygen level (predicted cell surface pO_2_ 5–10 mmHg) for maximizing infection. More extreme levels of hypoxia (predicted cell surface pO_2_<5 mmHg) resulted in increased late-stage parasite survival but arrested parasite development. The effects of hypoxia on late-stage EEF survival, but not EEF development, appear to be regulated in part by host-dependent HIF-1α mechanisms.

Establishing an *in vitro* model of liver-stage malaria has been an ongoing challenge for the field, due in part to the relatively poor maintenance of hepatic functions by existing culture platforms. With the development of the PHH MPCC system, it is now possible to achieve robust liver-stage malaria infection *in vitro* (March et al., 2013), but further optimization of infection efficiency remains advantageous. Our mathematical model predicts that conventional MPCCs are hyperoxic under conventional culture conditions, with estimated cell surface pO_2_ ranging from 110 to 130 mmHg (Table 1), whereas *in vivo* oxygen tensions in the liver range from 30 to 75 mmHg (Wölfle et al., 1983; Kietzmann and Jungermann, 1997). We have previously shown that achieving more physiological replication of the *in vivo* environment can improve hepatocyte function and disease modeling capacity *in vitro* (Allen et al., 2005). Thus, we hypothesized that liver-stage malaria infection might be more robust *in vitro* in the presence of atmospheric hypoxia. Indeed, the current observations that the sizes of *P. berghei*, *P. yoelii* and *P. falciparum* EEFs increase in PHHs under hypoxia *in vitro* is consistent with previous observations that primary hepatocytes respond to physiologically relevant oxygen gradients imposed upon them *in vitro* to recapitulate *in vivo* zonation phenotypes that are otherwise not observed *in vitro* (Allen et al., 2005). The observation that *P. berghei* and *P. yoelii* demonstrate increased numbers of EEFs under hypoxia, but not *P. falciparum*, suggests that the kinetics and extent of exposure to hypoxia for increased survival of the human malaria parasite differs from the rodent malaria species. The finding that there is an optimum cell surface pO_2_ (5–10 mmHg) for liver-stage malaria infection *in vitro* is consistent with the histopathology findings from *P. yoelii*-infected mouse liver sections, which show that EEFs in the perivenous region, which has the lowest sinusoidal oxygen tension of 30 mmHg, are larger than those in the periportal region (Fig. 1). Intriguingly, this optimum range of cell surface pO_2_ for PHH infection *in vitro* is lower than the 30–75 mmHg (Wölfle et al., 1983) reported in hepatic sinusoids *in vivo*.

One possible reason for this discrepancy is due to a lower hepatocyte surface pO_2_ *in vivo* than that previously measured in the hepatic sinusoid. This could be due either to the unsteady perfusion of the liver, which arises from the pulsatile flow that has been observed *in vivo* (McCuskey et al., 1983), or the significant oxygen consumption by the endothelium *in vivo* (Santilli et al., 2000). This hypothesis is supported by the observations that liver sections obtained from mice perfused with Hypoxyprobe™ show significant Hypoxyprobe™ adduct accumulation in the pericentral regions (Arteel et al., 1995) and that Hypoxyprobe™ forms such adducts only at pO_2_<10 mmHg (Varghese et al., 1976).

A second possible reason is that the optimal *in vitro* pO_2_ for malaria infection could simply be different from *in vivo* hepatic pO_2_. This could be because our *in vitro* model is missing key *in vivo* microenvironmental cues (growth factor gradients and cycling insulin/glucagon metabolism) that might result in the necessity for more extreme pO_2_ perturbations to optimize malaria infection *in vitro*. This disparity is consistent with the fact that *in vitro* infections, although improved under hypoxia, still require much higher multiplicities of infection than *in vivo* infections. It is also possible that the *in vivo* pO_2_ is not necessarily optimal because blood stage malaria parasitemia in rodents can be further increased under atmospheric hypoxia that simulates high-altitude atmospheres (Hughes and Tatum, 1956a).

A third reason lies in the possibility that our mathematical model underestimates cell surface pO_2_ *in vitro* due to the assumption that only diffusion transports oxygen to the cell surface. Furthermore, our mathematical model assumes that hepatocytes exhibit a constant oxygen consumption rate (OCR) (Rotem et al., 1992; Yarmush et al., 1992), which can vary with species, donor, time in culture (Rotem et al., 1994; Bhatia et al., 1996) and culture parameters like density and co-culture cell type.

The finding that liver-stage malaria infection *in vitro* has an optimal oxygen tension is also consistent with the microaerophilic nature of the blood stages of *P. falciparum*, which exhibit a propensity for better growth *in vitro* under ambient hypoxia (Trager and Jensen, 1976; Briolant et al., 2007), and in fact demonstrate optimum growth at an *in vitro* pO_2_ (2–3%, 15–25 mmHg) (Scheibel et al., 1979) that is lower than *in vivo* pO_2_ levels in the blood (4–13%, 30–100 mmHg) (Tsai et al., 2003). To extrapolate our findings to other *in vitro* liver-stage models, the appropriate atmospheric pO_2_ should be determined within a similar mathematical framework as described for MPCCs and take into account culture parameters such as effective hepatocyte density and oxygen diffusion distance (height of medium).

The beneficial effect of hypoxia on *in vitro* liver-stage malaria infection could be due to changes in the host cell that increase host cell susceptibility to initial parasite invasion or that favor parasite survival or development, or to changes in the parasite itself that promotes its own ability to survive and thrive in the host cell. Sporozoite entry assays (Fig. 4E) and infection of hepatocytes exposed to hypoxia only prior to invasion but not after infection (Fig. 4C) suggest that hypoxia does not increase hepatocyte susceptibility to sporozoite infection. Nonetheless, gene set enrichment analysis of PHH MPCCs incubated at ambient hypoxia versus normoxia showed a marked enrichment for the expression of HIF-1α related genes in hypoxic MPCCs (supplementary material Fig. S6A). HIF-1α plays a major role in the induction of cellular responses that mediate the adaptation of the host cell to hypoxic conditions. This response includes an increased expression of glucose transporters and multiple enzymes responsible for a metabolic shift towards anaerobic glycolysis (Warburg effect), as well as the downregulation of mitochondrial respiration. The latter in turn reduces mitochondrial oxygen consumption and the resultant generation of reactive oxygen species that occurs due to inefficient electron transport under hypoxic conditions (Weidemann and Johnson, 2008; Semenza, 2012). Among other *Apicomplexan* infections, host HIF-1α has been shown to be essential for *Toxoplasma gondii* survival and growth in host cells cultured at physiological oxygen levels (3% O_2_) (Spear et al., 2006), and is also necessary for the maintenance of *Leishmania amazonensis* parasitemia in human macrophages *in vitro* (Degrossoli et al., 2007). In fact, *Toxoplasma* and *Leishmania* infection increase HIF-1α protein levels as well as HIF-1α-regulated expression of glycolytic enzymes and glucose transporters (Spear et al., 2006; Degrossoli et al., 2007), suggesting that these *Apicomplexan* parasites actively activate host HIF-1α, presumably to favor their survival or growth.

Pharmacological activation of host HIF-1α in infected MPCCs by CoCl_2_ and DMOG increased EEF survival (Fig. 5B,D), but did not increase the EEF size distributions (Fig. 5C,E), suggesting that the effects of ambient hypoxia on liver-stage malaria EEF numbers and EEF sizes are driven by distinct mechanisms, with host HIF-1α playing a role in maintaining the survival of EEFs but not necessarily driving EEF growth. This hypothesis is supported by the observations that the total number of EEFs increased monotonically with decreasing cell surface pO_2_ (Fig. 3E) but the number of well-developed EEFs exhibited a biphasic relationship with decreasing cell surface pO_2_ (Fig. 3D). However, in the absence of genetic perturbation of host HIF-1α, the possibility that hypoxia, CoCl_2_ or DMOG impact alternative pathways in the parasite that mediate the observed infection phenotype cannot be excluded.

One possible mechanism that could explain the effect of hypoxia on EEF size is the activation of the AMPK pathway in the host cell. AMPK activation is known to induce autophagy in mammalian cells (Liang et al., 2007; Kim et al., 2011), whereas autophagy of *Plasmodium* EEFs in human hepatoma cells is known to occur and might be necessary for the growth of *Plasmodium* EEFs (Eickel et al., 2013). AMPK activation also mediates mitophagy and mitochondrial biogenesis (Mihaylova and Shaw, 2011), which results in increased mitochondrial renewal and might promote *Plasmodium* EEF development. In support of this hypothesis, *Toxoplasma gondii*, another *Apicomplexan* parasite, is known to tether host mitochondria to its parasitophorous vacuole membrane (Sinai and Joiner, 2001), suggesting that host mitochondria is necessary for *Toxoplasma* growth in the host cell.

In addition to host-mediated mechanisms, the malaria parasite might contain either oxygen sensors that directly respond to hypoxia or indirect mechanisms that limit their ability to respond to oxidative stress. It is difficult to distinguish the parasite-specific and the host-specific responses to hypoxia. For example, intraerythrocytic *P. falciparum* is heavily dependent on antioxidant systems despite its almost totally fermentative lifestyle, yet it lacks significant antioxidant enzymes like catalase and glutathione peroxidase, which play major protective roles in mammalian cells (Müller, 2004; Vonlaufen et al., 2008). This suggests that the *Plasmodium* liver stage might also be predisposed to being overwhelmed by environmental oxidants and that hypoxia might reduce the energy expenditure for the maintenance of redox balance in the EEF.

A caveat of our findings is that changes in atmospheric oxygen could result in modulations beyond simply adjusting cell surface oxygen levels. The modulation of hepatocyte metabolism under hypoxia might result in different rates of nutrient consumption and waste generation, which could lead to secondary effects like changes in pH. This study also does not specifically identify the role of the co-culture nonparenchymal cell type in the infection phenotype, and does not use a liver-derived nonparenchymal cell type like sinusoidal endothelial cells or Kupffer cells. The *in vivo* histopathology findings are correlative and not causal, as the presence of an oxygen gradient along the sinusoid is only one of many other gradients that simultaneously exist in the liver. Thus, it is challenging to decisively untangle the various contributions of oxygen gradients in our observations, but oxygen is more likely to be the driver of these other sinusoidal gradients than vice versa. More work is required to characterize the role of HIF-1α on *Plasmodium* infection of PHHs, including performing siRNA-mediated knockdown and overexpression of HIF-1α in primary hepatocytes *in vitro*, or using a HIF-1α knockout mouse. Furthermore, the downstream mechanisms of HIF-1α that are ultimately responsible for the effect of hypoxia on *Plasmodium* infection of PHHs remain to be uncovered. These mechanisms could include increases in glycolysis or iron uptake by hepatocytes, which could lead to an elevation in intracellular glucose or iron levels that are accessible to the *Plasmodium* EEF. Other mechanisms that could contribute to the effect of hypoxia on infection include AMPK activation in host cells, leading to a starvation response that decreases intracellular ROS levels and frees up resources for the malaria EEF.

In an era of a renewed effort towards global malaria eradication, the finding that oxygen levels influence *in vitro Plasmodium* liver-stage infection of PHHs, in combination with existing literature on the impact of oxygen on the maintenance of *in vivo*-like hepatocyte functions *in vitro*, highlights the importance of optimizing oxygen levels experienced by PHHs *in vitro* so as to develop improved *in vitro* models of liver-stage malaria for antimalarial drug development.

## MATERIALS AND METHODS

### Reagents and cell culture

Dimethyloxalylglycine (DMOG) was obtained from Cayman Chemicals (Ann Arbor, MI), and cobalt(II) chloride was obtained from Sigma (St Louis, MO). Cryopreserved PHHs were purchased from vendors permitted to sell products derived from human organs procured in the United States by federally designated Organ Procurement Organizations. CellzDirect (Invitrogen, Grand Island, NY) was the vendor used in this study. Human hepatocyte culture medium was high glucose Dulbecco’s modified Eagle’s medium (DMEM) with 10% (v/v) fetal bovine serum (FBS), 1% (v/v) ITS™ (BD Biosciences), 7 ng/ml glucagon, 40 ng/ml dexamethasone, 15 mM HEPES, and 1% (v/v) penicillin-streptomycin. J2-3T3 murine embryonic fibroblasts (a gift of Howard Green, Harvard Medical School) were cultured at <15 passages in fibroblast medium comprising DMEM with high glucose, 10% (v/v) bovine serum and 1% (v/v) penicillin-streptomycin.

### MPCCs of primary human hepatocytes and supportive stromal cells

Coverslips (12 mm) that were placed into tissue culture polystyrene 24-well plates or glass-bottomed 96-well plates were coated homogenously with rat tail type I collagen (50 μg/ml) and subjected to soft-lithographic techniques (Khetani and Bhatia, 2008) to pattern the collagen into micro-islands (of diameter 500 μm) that mediate selective hepatocyte adhesion. To create MPCCs, cryopreserved PHHs were thawed and pelleted by centrifugation at 100×***g*** for 6 minutes, assessed for viability using Trypan Blue exclusion (typically 70–90%), and then seeded on collagen-micropatterned plates in DMEM. The cells were washed with DMEM 2–4 hours later and replaced with human hepatocyte culture medium. 3T3-J2 murine embryonic fibroblasts were seeded (40,000 cells in each well of a 24-well plate and 7000 cells in each well of a 96-well plate) in human hepatocyte medium 3 h after *Plasmodium* sporozoite infection. Medium was replaced every 24 hours.

### Sporozoites

*P. berghei ANKA* and *P. yoelii* sporozoites were obtained by dissection of the salivary glands of infected *Anopheles stephensi* mosquitoes obtained from the insectaries at New York University (New York, NY) or Harvard School of Public Health (Boston, MA). *P. falciparum* sporozoites were obtained by dissection of the salivary glands of infected *Anopheles gambiae* mosquitoes obtained from the insectary at Johns Hopkins School of Public Health (Baltimore, MD).

### Infection of MPCCs

*P. berghei*, *P. yoelii* or *P. falciparum* sporozoites from dissected mosquito glands were centrifuged at 3000 rpm for 5 minutes onto micropatterned primary hepatocytes cultured without fibroblasts for 2 or 3 days before infection at a multiplicity of infection of 1 to 3. After incubation at 37°C and 5% CO_2_ for 3 hours, the wells were washed twice and J2-3T3 fibroblasts were added to establish the MPCCs. Media was replaced daily. Samples were fixed at 48, 56 or 65 hours post-infection with *P. berghei* and *P. yoelii*, and 4 or 6 days post-infection with *P. falciparum*.

### Immunofluorescence assay

Infected MPCCs were fixed with −20°C methanol for 10 minutes at 4°C, washed thrice with PBS, blocked with 2% BSA in PBS for 30 minutes and then incubated for 1 hour at room temperature with a primary antibody mouse anti-PbHSP70 (clone 2E6; 1:200 for *P. berghei* and *P. yoelii*), rabbit anti-PbMSP1 (1:500 for *P. berghei*) or mouse anti-PfHSP70 (clone 4C9, Sanaria; 1:200 for *P. falciparum*). Samples were washed thrice with PBS before incubation for 1 hour at room temperature with secondary antibody: goat anti-mouse conjugated to Alexa Fluor 594 or Alexa Fluor 488 or donkey anti-rabbit conjugated to Alexa Fluor 488 (1:400; Invitrogen). Samples were washed thrice with PBS, with nuclei counterstained with Hoechst 33258 (1:1000; Invitrogen), and then mounted on glass slides with Fluoromount G (Southern Biotech, Birmingham, AL). For samples in 96-well plates, 50 μl of Aquamount (Thermo-Scientific, West Palm Beach, FL) was added per well after counterstaining with Hoechst. Images were captured on a Nikon Eclipse Ti fluorescence microscope.

### Sporozoite gliding assay

Motility of cryopreserved sporozoites was determined in each batch to define the number of infective sporozoites. Sporozoite gliding was evaluated with 30,000 sporozoites for 40 minutes in complete DMEM, at 37°C on glass cover slips pre-coated for 1 hour at 37°C with an antibody against *P. berghei* circumsporozoite protein (PbCSP) (clone 3D11, 10 μg/ml). Sporozoites were subsequently fixed in 4% paraformaldehyde (PFA) for 10 minutes and stained with anti-PbCSP antibody. The percentage of sporozoites associated with CSP trails was visualized by fluorescence microscopy. Quantification was performed by counting the average percentage of sporozoites that perform at least one circle.

### Double-staining assay for sporozoite entry

At 3 hours post-infection, MPCCs were fixed and stained using a double-staining protocol as previously described (Rénia et al., 1988). Briefly, to label extracellular sporozoites, the samples were first fixed with 4% paraformaldehyde for 10 minutes at room temperature, blocked with 2% BSA in PBS, incubated with a primary mouse anti-PbCSP (clone 3D11, 10 μg/ml), washed thrice in PBS and incubated with a secondary goat anti-mouse Alexa Fluor 488 conjugate. This was followed by a permeabilization with −20°C methanol for 10 minutes at 4°C, incubation with the same primary mouse anti-PbCSP, washing thrice with PBS, and incubation with a secondary goat anti-mouse Alexa Fluor 594 conjugate. This second step labels both intracellular and extracellular sporozoites. The samples were counterstained with Hoechst and mounted on glass slides as described above. The number of invaded sporozoites (stained green only) in PHHs was quantified.

### Gene expression microarray analysis

MPCCs established from two different donor lots of PHHs were incubated under ambient hypoxia overnight (18–24 hours), and total RNA was extracted using TRIZOL and a Qiagen RNA clean-up kit. The RNA was analyzed using a Bioanalyzer before being labeled with Cy 3 and Cy 5 for the normoxic versus hypoxic samples, respectively. The labeled RNA from biological triplicates was loaded onto an Agilent (Santa Clara, CA) SurePrint G3 Human Gene Expression Microarray. The microarray data was analyzed by performing a Gene Set Enrichment Analysis (GSEA), which determines whether a predefined set of genes shows statistically significant differences between two biological conditions (Subramanian et al., 2005), applying a false discovery rate of 25%.

### Mathematical model

To estimate the cell surface oxygen tensions in MPCCs, the transport and consumption of oxygen was modeled as a one-dimensional reaction-diffusion system, as described previously (Yarmush et al., 1992). The average number of hepatocytes per hepatocyte island in the MPCCs was determined by manual counts with light microscopy. The following assumptions were made in applying this model. First, the oxygen consumption rate of primary rat hepatocytes was used due to absence of the oxygen consumption rates of PHHs. Second, as the oxygen consumption rate of fibroblasts is only one-tenth that of primary rat hepatocytes and the oxygen consumption rate of random co-cultures of hepatocytes and fibroblasts is similar to that of hepatocytes alone (Allen et al., 2005), the oxygen consumption of MPCCs was assumed to be that of hepatocytes alone. Third, the oxygen consumption rates were assumed to be independent of culture format and constant throughout the infection experiments.

### Hypoxyprobe™ assay

Hypoxyprobe™ (pimonidazole hydrochloride, Burlington, MA) forms covalent adducts in hypoxic cells at cell surface pO_2_<10 mmHg (Varghese et al., 1976) and was used as a hypoxia marker in PHHs. Hypoxia was first induced in primary hepatocytes by atmospheric hypoxia, variation of the height of the medium or variation in hepatocyte island densities. Pimonidazole hydrochloride was then added from a 200 mM stock solution (constituted in PBS) into the culture medium (without changing medium to avoid disturbing the steady-state oxygen gradient) at a 1:1000 dilution to achieve a final working concentration of 200 μM. Cells were incubated at 37°C for 2 hours, washed twice with PBS and fixed with chilled methanol for 10 minutes at 4°C. Adduct formation was detected by direct immunofluorescence using the HP-Red549 antibody (Hypoxyprobe™) at a 1:100 dilution.

### Histological analysis

Liver slices (50 μm) were obtained from C57BL/6 mice (Charles River, Wilmington, MA) at 46 hours post-infection with GFP-expressing *P. yoelii* sporozoites. Maximal EEF size of EEFs in the periportal area (up to eight hepatocytes wide, from portal vein) and in the centrilobular area (up to eight hepatocytes wide, from central vein) were measured using *z*-stacks of these EEFs acquired via confocal imaging (Olympus, Center Valley, PA).

### Statistics

Experiments were repeated three or more times with triplicate samples for each condition. Data from representative experiments are presented, and similar trends were seen in multiple experiments. Two-tailed *t*-tests were performed for all comparisons between two conditions (e.g. 21% versus 4% O_2_) at a single time point. One way ANOVAs were performed for comparisons involving three or more conditions (e.g. 21% versus different periods of 4% O_2_) at a single time point with Tukey’s post-hoc test for multiple comparisons. Two way ANOVAs were performed for comparisons involving both simultaneous variation in time points post-infection and oxygen level (e.g. 21% versus 4% O_2_ at 48, 56 and 65 hours post-infection for *P. berghei*) with Bonferroni’s post-hoc test for multiple comparisons. All error bars represent s.e.m.
